# Characterization of a Riboflavin-Producing Mutant of *Bacillus subtilis* Isolated by Droplet-Based Microfluidics Screening

**DOI:** 10.3390/microorganisms11041070

**Published:** 2023-04-20

**Authors:** Fan Xu, Chuan Liu, Miaomiao Xia, Shixin Li, Ran Tu, Sijia Wang, Hongxing Jin, Dawei Zhang

**Affiliations:** 1 School of Chemical Engineering, Hebei University of Technology, Tianjin 300131, China; 2 Tianjin Institute of Industrial Biotechnology, Chinese Academy of Sciences, Tianjin 300308, China; 3 Key Laboratory of Systems Microbial Biotechnology, Chinese Academy of Sciences, Tianjin 300308, China; 4 University of Chinese Academy of Sciences, Beijing 100049, China; 5 School of Biological Engineering, Tianjin University of Science and Technology, Tianjin 300222, China; 6 School of Biological Engineering, Dalian Polytechnic University, Dalian 116034, China

**Keywords:** Bioengineering, fermentation, Molecular biology, high-throughput screening, riboflavin, *Bacillus subtilis*, droplet-based microfluidics

## Abstract

*Bacillus subtilis* is one of the commonly used industrial strains for riboflavin production. High-throughput screening is useful in biotechnology, but there are still an insufficient number of articles focusing on improving the riboflavin production of *B. subtilis* by this powerful tool. With droplet-based microfluidics technology, single cells can be encapsulated in droplets. The screening can be carried out by detecting the fluorescence intensity of secreted riboflavin. Thus, an efficient and high-throughput screening method suitable for riboflavin production strain improvement could be established. In this study, droplet-based microfluidics screening was applied, and a more competitive riboflavin producer U3 was selected from the random mutation library of strain S1. The riboflavin production and biomass of U3 were higher than that of S1 in flask fermentation. In addition, the results of fed-batch fermentation showed that the riboflavin production of U3 was 24.3 g/L, an 18% increase compared with the parent strain S1 (20.6 g/L), and the yield (g riboflavin/100 g glucose) increased by 19%, from 7.3 (S1) to 8.7 (U3). Two mutations of U3 (*sinR^G89R^* and *icd^D28E^*) were identified through whole genome sequencing and comparison. Then they were introduced into BS168DR (parent of S1) for further analysis, which also caused riboflavin production to increase. This paper provides protocols for screening riboflavin-producing *B. subtilis* with droplet-based microfluidics technology and reveals mutations in riboflavin overproduction strains.

## 1. Introduction

Riboflavin, also known as vitamin B2, is one of the trace elements necessary to maintain human life metabolism. It was first discovered in milk by Blythto in 1879 [1], and in 1933, it was successfully purified and named riboflavin by Paul György, Richard Kuhn, and Theodore Wagner-Jauregg [2]. Now riboflavin has become a large-scale chemical widely used in medical, food, feed, and other fields [3,4]. At first, *Ashbya gossypii* was used in the production of riboflavin by microbial fermentation. Later, the genetic modification technology of *Bacillus subtilis* was gradually improved, and *B. subtilis* became the main strain for the production of riboflavin by microbial fermentation in bulk amounts [5,6]. Although the industrial production process of riboflavin is becoming mature, there is still room for improvement in production. In the field of mutation breeding, Hu Jianhua et al. studied the heavy ion radiation mutation breeding technology [7]. Zhu Jinshan et al. used the atmospheric pressure room temperature plasma (ARTP) mutation technology for breeding [8], both of which have improved the production of riboflavin by *B. subtilis* to varying degrees, but there are few reports of the application of high-throughput screening in strain breeding, although this screening method is significant to improve the efficiency of selection.

Droplet-based microfluidics technology is a developing method for high-throughput screening, which has the advantages of saving time, space, and cost in the experiment. The dispersed and independent droplets are suitable for encapsulating single cells for subsequent screening experiments [9,10]. Riboflavin-producing *B. subtilis* is ideal for droplet-based microfluidics screening because secreted riboflavin can be measured by the fluorescence detector. However, the percentage of survival cells is lower than expected [11].

In the previous work, the strain was finally screened by the 24-well plate rather than the droplet-based microfluidics because only a few clones were obtained after droplet separation. In this study, a new protocol of droplet preparation and separation was established based on the research by Fu et al. [11] with many modifications. Riboflavin production strain S1 [11], obtained by random mutation from BS168DR [12], was selected as the parent strain. A random mutagenic library of S1 was constructed, and strain U3 was screened out, then its riboflavin production was evaluated by fermentation. The results showed that its riboflavin production was increased compared with that of S1, and a higher concentration of residual glucose was demanded in fed-batch fermentation. Two mutation sites: *sinR^G89R^* and *icd^D28E^*, were identified through whole genome sequencing and comparison. Their functions of them were verified in the parent strain BS168DR, which confirmed the effect of the mutation sites and provided guidance for the subsequent genetic engineering.

## 2. Materials and Methods

### 2.1. Strains, Medium, Reagents, and Instruments

All strains used in this study are described in Table S1. S1 is a riboflavin-producing *B. subtilis* mutated from BS168DR, and BS168DR used in this experiment is a genetic strain modified based on *B. subtilis* 168. The random mutation library of the S1 strain was obtained by ARTP mutation. Strains U1-U8 were mutants obtained from droplet-based microfluidics screening. sinRm, and icdm are single-site mutation strains engineering from BS168DR.SinRm-CR and icdm-CR were strains with cat-araR (CR) fragment insertion in the engineering process. The strain named sim comprised mutations of *sinR* and *icd*.

LB medium (L^−1^): 10 g tryptone, 5 g yeast extract, 10 g sodium chloride.

YP medium (L^−1^): 5 g corn syrup dry powder, 10 g sucrose, 5 g magnesium sulfate, 5 g yeast extract, 3 g Dipotassium hydrogen phosphate, 2 g potassium dihydrogen phosphate. The initial pH of the medium was adjusted to 7.2 with NaOH before sterilization.

Fermentation medium (L^−1^): 20 g corn syrup dry powder, 3 g yeast powder, 1 g magnesium sulfate, 1.5 g potassium dihydrogen phosphate, 0.5 g potassium dihydrogen phosphate, 1.7 g Betaine. The initial pH of the medium was adjusted to 7.2 with NaOH before sterilization.

Feeding medium (L^−1^): 550 g glucose monohydrate, 15 g corn syrup dry powder, 0.3 g potassium dihydrogen phosphate.

Biofilm detection medium(L^−1^): 50 g Glycerol, 50 g yeast extract, 50 g soy peptone, 3.8 g dipotassium hydrogen phosphate, 1.6 g potassium dihydrogen phosphate.

Main reagent manufacturers: Tryptone (Vicbio Biotechnology Co., Ltd., Beijing, China), yeast extract (Vicbio Biotechnology Co., Ltd., Beijing, China), sodium chloride (Sangon Biotech (Shanghai) Co., Ltd., Shanghai, China), corn syrup dry powder (Beijing Hongrun baoshun Technology Co., Ltd., Beijing, China), sucrose (Samyang Co., Ltd., Seoul, Korea), magnesium sulfate (Sangon Biotech (Shanghai) Co., Ltd., Shanghai, China), dipotassium hydrogen phosphate (Beijing Solarbio Technology Co., Ltd., Beijing, China), potassium dihydrogen phosphate (Beijing Solarbio Technology Co., Ltd., Beijing, China), betaine (Shanghai Macklin Biochemical Co., Ltd., Shanghai, China), glucose monohydrate (Tianjin Yongda Chemical Reagent Co., Ltd., Tianjin, China), HFE-7500 fluorinated oil (3M Co., Ltd., Shanghai, China), 2% surfactant (RAN Biotechnology Co., Ltd., Shanghai, China).

Main instrument manufacturers: bioflo310 bioreactor (7.5 L) (Eppendorf (Shanghai) Co., Ltd., Shanghai, China), MAX300-LG gas analysis systems (Process Insights (Suzhou) Co., Ltd., Suzhou, China), SBA-40E biosensor (Jinan YanHe biotechnology Co., Jinan, China), FE28-Standard pH meter (Mettler-Toledo (China), Inc., Shanghai, China). ARTP-II type ARTP mutagenic instrument (Beijing Siqingyuan Biotechnology Co., Beijing, China). UV-1000 Spectrophotometer (AoYi instrument shanghai Co., Ltd., Shanghai, China)

### 2.2. Gene Recombination Method

In this experiment, the double exchange homologous recombination method was used to construct engineering strains [13]; all primers used in this study are listed in Table S2. The specific steps are as follows:

1. The upstream homologous arm (UP) and downstream homologous arm (DN) of the target gene were amplified using the *B. subtilis* 168 (BS168) genome as the template, and the fragment containing the screening marker cat-araR (CR) and the direct repeats (DR) was amplified using the strain with CR as the template, and the corresponding mutation site was introduced in the design of the primer.
2. Upstream, CR, and downstream fragments could be overlapped to generate the UP-CR-DN fragment. The product was transferred into the *B. subtilis* by the Spizizen transformation method. The cultures were spread on the LB solid plate containing 10 μg/mL chloramphenicol.
3. Based on the principle of homologous recombination, the homologous sequence was integrated into the genome of *B. subtilis*. The positive clones can be screened through chloramphenicol resistance, and the colonies can be verified.
4. The positive clones were inoculated into the test tube containing 10 μg/mL chloramphenicol 5 mL LB medium, incubated at 37 °C, 200 r/min for 12 h. The strain with CR fragment as a positive screen mark was stored at −80 °C.
5. The cultures from the previous test tube were inoculated into the test tube containing 5 mL of LB medium without resistance and incubated at 37 °C for 8 h. Because of the existence of the same short sequence DR, there will be secondary homologous recombination in the cells, and the screening marker CR will be discarded. Then the cultures were spread on the LB solid plate containing 60 μg/mL neomycin, and the positive clones could be screened.
6. PCR verification was carried out, and the single colony with the correct band size was inoculated into the test tube containing 5 mL LB medium with 20 μg/mL neomycin at 37 °C, 200 r/min for 12 h, and the strain was stored at −80 °C.

### 2.3. Droplet Preparation and Mutagenesis

A single colony of the S1 was streaked onto an LB slope medium and incubated at 37 °C for 48 h. The cultures from the slope medium were inoculated into a 500 mL flask containing 80 mL of YP medium, shaken at 37 °C, and 200 r/min for 12 h until OD_600_ increased to 10–12. The cultures from the YP medium were inoculated into the fermentation medium, shaken at 37 °C, and 200 r/min. When the glucose concentration was below 10 g/L, the cells were collected by centrifugation and resuspended in fresh fermentation medium. The ARTP mutagenesis was carried out [14]. The working radiofrequency power input and gas flow rate were set to 100 W and 10.0 SLM (standard liter per minute), respectively. The bacterial samples were treated for 30 s. The mutated cells were collected and adjusted to OD_600_ = 0.5 with fermentation medium, and then the cells were encapsulated with droplet-based microfluidics. The suspended bacterial solution used as the water phase was injected into the chip water phase inlet at a flow rate of 200 μL/h. HFE-7500 fluorinated oil containing 2.0% (*w*/*w*) surfactant was used as the oil phase and was injected into the oil phase inlet of the chip at a flow rate of 400 μL/h. Because of the shearing action of the oil phase, water-in-oil droplets are generated, and the diameter of the droplets is about 25 μm.

### 2.4. Droplet Separation

The droplets were incubated at 37 °C for 4 h when the fluorescence could be detected, and droplet separation was carried out by the microfluidic droplet sorting device [15,16]. During separation, the droplets were re-injected into the droplet phase inlet of the separation chip at a flow rate of 12 μL/h. HFE-7500 was used as spacer oil and injected into the oil phase inlet of the sorting chip at a flow rate of 400 μL/h. Observe with an inverted microscope with a 20-fold objective, and a 488 nm wavelength laser was used as excitation light. Align the laser point with the detection point. Under the action of the laser, the fluorescent substance (riboflavin) in the droplet was excited and emitted fluorescence. The fluorescence passes through the dichroic mirror, which filters the emitted light of other wavelengths except for the 520 nm emission light. Then it was detected by a photomultiplier tube (PMT), which transformed and amplified the weak fluorescence signal into an electric signal. After the acquisition card was collected and processed the information, the fluorescence signal was shown on the software through the peak spectrum. The high voltage amplifier sent out a voltage of 500 V after setting the threshold (first 2%), and its electric field forced the target droplets with the required fluorescence intensity to deflect into the separation channel, which was collected in a 1.5 mL centrifuge tube, while the non-target droplets are discharged through the waste liquid channel. The average sorting frequency is 350 Hz. After encapsulation, the liquid drops were spread on LB solid medium plate and incubated at 37 °C for 12–36 h.

### 2.5. Flask Fermentation Conditions, Measurement of Cell Density, Riboflavin Titers, and Concentration of Glucose and Sucrose

The bacterial solution from the frozen storage tube was spread on the LB solid plate with corresponding resistance and incubated at 37 °C for 24 h. The cultures were scraped into YP medium and inoculated into a 500 mL shake flask containing 80 mL YP medium with initial OD_600_ = 0.1, incubated at 37 °C, 200 r/min for 48 h. Samples were diluted with 0.9% sodium chloride solution at an appropriate multiple to make the OD_600_ within the range of 0.2 to 0.8 and measure the growth of the bacteria with a spectrophotometer. Determination of riboflavin: The sample was diluted with 0.01 mol/L NaOH solution, dissolved for 20 min, then centrifuged at 12,000 r/min for 3 min. The supernatant was taken to determine the absorbance value at 444 nm, and the riboflavin production was calculated with the formula: production (mg/L) = (absorbance × Dilution ratio)/0.0321 [17]. The glucose and sucrose were determined by the SBA-40E biosensor, as described previously [18]. Three replicates were examined, and the data were analyzed by the Student’s *t*-test.

### 2.6. Fed-Batch Fermentation

A single colony of the S1 was streaked onto an LB slope medium and incubated at 37 °C for 48 h. To prepare the seed, the cultures from the slope medium were inoculated into a 500 mL flask containing 80 mL YP medium with the corresponding resistance and incubated at 37 °C for 48 h. Then the seed was inoculated into the bioreactor containing the fermentation medium at a ratio of 15% (*v*/*v*). The fermentation condition was maintained at 40 °C, pH = 7.2, and the fermentation period was about 46 h. The feeding strategy was as follows: the amount of glucose (g) needed to be fed in the next period = the amount of glucose (g) fed in the latest period × [(3 × OD_600_ of the present time point-OD_600_ of the latest time point) × 2/(OD_600_ of present time point + OD_600_ of the latest time point)] + (glucose concentration (g/L) of the latest time point × volume (L) of the latest time point-glucose concentration (g/L) of the present time point × volume (L) of the present time point) × 2.

### 2.7. Biofilm Determination

*B. subtilis* was cultured in a 24-well plate in a biofilm detection medium and incubated at 37 °C for 48 h. The ability of biofilm formation was determined by crystal violet staining assays [19]. The supernatant was removed, and 400 μL 0.01% crystal violet solution (CV) was added into each hole, then it was incubated at room temperature for 20 min. The sediment was washed with phosphate-buffered saline (PBS) three times and dried at 55 °C, then 400 μL 33% acetic acid was added to each hole for decolorization. Finally, the absorbance was measured at 570 nm by spectrophotometer. Three replicates were examined, and the data were analyzed by the Student’s *t*-test.

## 3. Results

### 3.1. Strain Screening

The preculture and incubation process before screening was simulated and optimized according to our fed-batch fermentation procedure. The method for preparation of S1 culture on LB solid medium and LB slope medium was mentioned in material and method 2.3. The cultures were inoculated into YP medium and shaken at 37 °C, 200 rpm for 12 h (until the post-exponential growth phase). Then the precultures in the YP medium were inoculated into the fermentation medium at a ratio of 5% (*v*/*v*) and incubated at 37 °C, 200 rpm. When the concentration of residual glucose was below 10 g/L, cells were washed with fresh fermentation medium (20 g/L glucose) for simulated feeding steps in the fed-batch procedure. Then the cell density was adjusted to OD_600_ = 1, and ARTP mutagenesis was carried out. The mutated cells were collected and resuspended with fermentation medium to make the OD_600_ = 0.5, and then the cells were encapsulated with droplet-based microfluidics. The time for the cultivation of the droplet in the incubator was reduced to 4 h compared with the previous protocol, and then the droplet separation was carried out.

**Figure 1:**
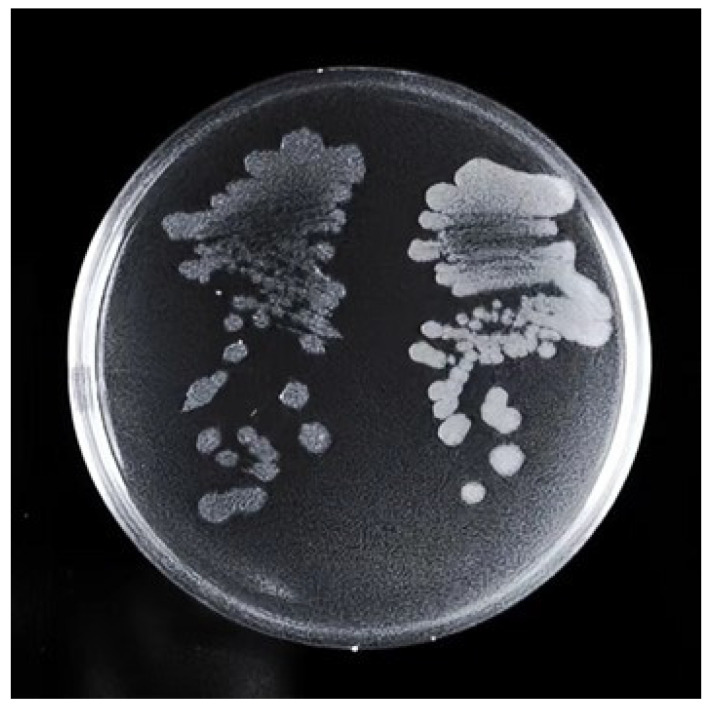
Comparison of colony morphology between S1 (**left**) and U3 (**right**). Strains were streaked on LB solid medium plate and incubated at 37 °C for 36 h.

About 15,000 fluorescent droplets were screened by droplet-based microfluidics fluorescence screening, the droplets with the first 2% fluorescence intensity were screened, and a total of eight mutagenic strains named U1-U8 were obtained. It was found that the colony morphology of U3 was significantly different from that of the S1 strain (Figure 1). It showed that the U3 colony was thicker than the S1 colony. The edge of the S1 colony was fuzzy, while the edge of the U3 colony was clearer and cleaner. When scraping, the U3 colony was more dry, fragmented, and hard to dissolve.

### 3.2. Production of Riboflavin of Mutants in Flask and Fed-Batch Fermentation

**Figure 2:**
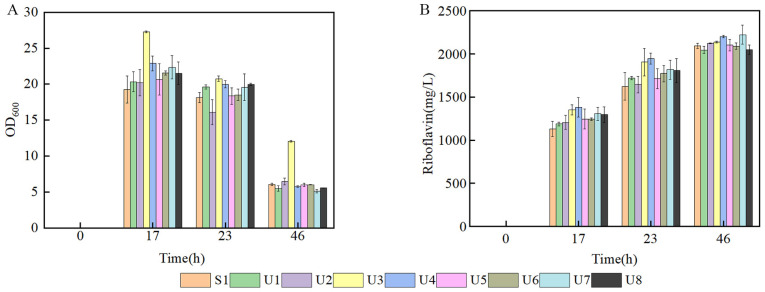
Batch fermentation by strains S1 and U1-U8 in 500 mL shake flasks containing 80 mL of YP medium. (**A**) time course profile of cell growth; (**B**) riboflavin production during fermentation.

In order to investigate the production of different mutant strains; flask fermentation was carried out in a YP medium at 37 °C for 46 h. The biomass of U3 was always the highest compared with the control strain and other mutant strains, indicating that the characteristic of U3 growth was greatly changed. The maximal OD_600_ of U3 reached 27.29 at 17 h. (Figure 2A). The production of U3 was 2138.32 mg/L at 46 h, while the control strains produced 2093.46 mg/L riboflavin (Figure 2B).

**Figure 3:**
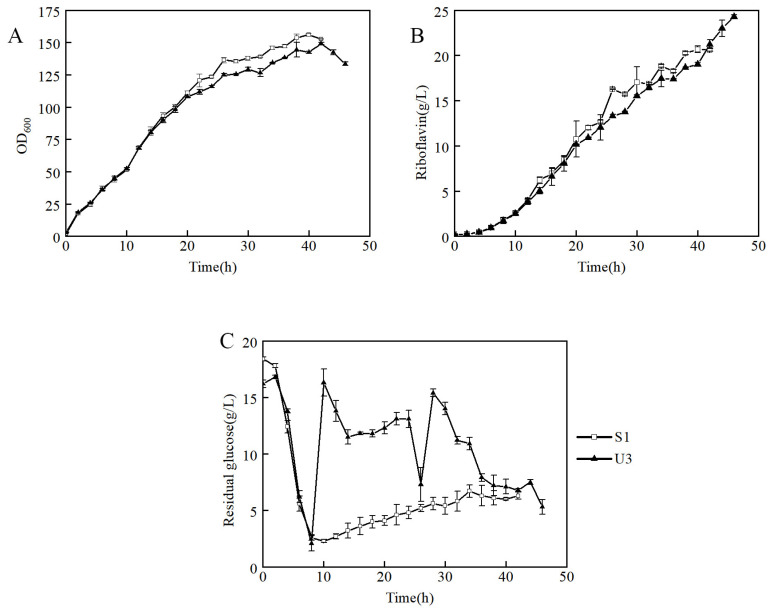
Fed-batch fermentation by strains S1 and U3 in a 7.5-L bioreactor containing 3 L of fermentation medium. (**A**) time course profile of cell growth; (**B**) riboflavin production during fermentation; (**C**) concentration of residual glucose.

To further investigate the ability to produce riboflavin of U3, the U3 and S1 strains were inoculated into a 7.5-L bioreactor for fed-batch fermentation in the fermentation medium. It showed that the growth curves of U3 and S1 were similar before 20 h, and then the biomass of U3 was slightly lower than that of S1 (Figure 3A). The production of U3 was lower at the early and middle stages but exceeded that of S1 at 42 h and showed a continuously rising trend (Figure 3B).

It indicated that strain U3 has three distinct characteristics from that of S1: (1) At 6–8 h, the initial glucose of S1 and U3 was consumed, so the feeding was initiated. During the beginning of the feeding process, the dissolved oxygen of the S1 strain decreased rapidly after feeding and then remained relatively stable. However, the dissolved oxygen of U3 did not decrease significantly after feeding. The problem was solved when the residual glucose was maintained at a high level (Figure 3C). It indicated that the U3 strain had a higher demand for residual glucose than the S1 strain. (2) The production of U3 and S1 were 24.3 g/L and 20.6 g/L respectively, and the production of U3 strain was 18% higher than that of S1. (3) The yields of U3 and S1 were 8.7% and 7.3%, respectively, and the yield of the U3 strain was 19% higher than that of S1. To sum up, it indicated that the U3 strain had a higher demand for residual glucose concentration and a higher riboflavin production capacity than S1.

**Figure 4:**
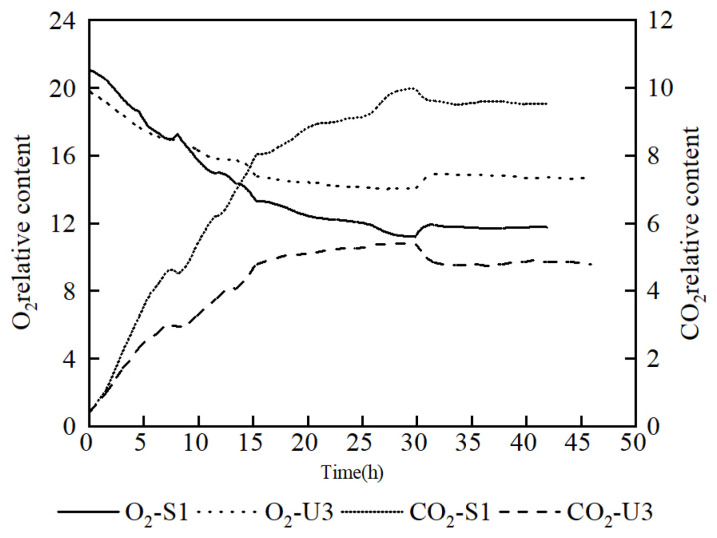
Comparison of off-gas analysis data between S1 and U3 on fed-batch fermentation.

In order to investigate the respiratory intensity in the fermentation process, an analysis of off-gas was carried out. It showed that the O_2_ content in the off-gas of U3 was lower than that of S1 at the early stage, but it exceeded that of S1 at nearly 8 h. At the same time, the CO_2_ content of U3 remained at a significantly lower level than that of S1 throughout the fermentation process, which proves that the respiratory intensity of U3 was weaker than that of S1 (Figure 4). It also indicated that the reduced CO_2_ emission was the reason for the increasing yield of U3.

### 3.3. Comparison and Analysis of the Genome Sequence of U3 with S1

In order to analyze the causes of these changes, the whole genome sequencing of the U3 strain was carried out and conducted further research on the mutations. The results of genome sequencing showed that there were two genes mutated in U3 compared with the S1 strain: *sinR* and *icd*. The mutation of G at 265 of *sinR* gene to A and the mutation of C at 84 of *icd* gene to A, resulting in the glycine at 89 of SinR changed to arginine, and the aspartate at 28 of Icd changed to glutamate, separately, which were not reported before.

Subtiwiki database shows that *sinR* is a transcriptional regulator (Xre family) of post-exponential-phase response genes. The regulation of biofilm synthesis is also one of the main functions of *sinR*. Biofilm is a dynamic collection composed of a variety of biological macromolecules, including stroma-producing cells, motion cells, and spore-forming cells [20]. These cells are surrounded by a matrix, including exopolysaccharides (EPS), fibrils formed by secreted protein (TasA), and extracellular DNA. In *B. subtilis*, EPS and TasA are encoded by the *epsA-C* operon and *yqxM tasA* operon, respectively [21,22]. The expression of the *yqxM tasA* operon is controlled by the regulatory repressor SinI and the repressor protein SinR [23].

The *icd* gene encodes isocitrate dehydrogenase in *B. subtilis*. The enzyme is in the tricarboxylic acid cycle (TCA cycle), and the deletion of *icd* will cause an accumulation of citrate [24]. Anyway, the *icd* gene also has the function of affecting sporulation formation. It was reported that the spore-forming ability of *B. subtilis* strains lacking *icd* was greatly reduced. Through the introduction and expression of the *Escherichia coli icd* gene, it can overcome the defects of growth and sporulation [25]. It was reported that sporulation formation was related to decreasing of GTP, which was the precursor of riboflavin [26]. To further investigate the effect of *sinR^G89R^* and *icd^D28E^* mutation, the mutation sites were reversely introduced into the parent strains for verification.

### 3.4. Reverse Verification of Mutation Site and Fermentation Results

Since the S1 strain inactivated the recombinant gene *recA* after mutation, it was difficult to introduce and verify the mutation site on the background of S1. BS168DR is the parent strain of S1 and is convenient for gene editing and has a clearer background. Thus, further verification was carried out in BS168DR. The mutation sites *sinR^G89R^* and *icd^D28E^* was introduced into the BS168DR strain by the double exchange homologous recombination method. This method of gene editing will produce a strain with a nearly 2000 bp fragment (CR) in the middle of the target gene. The strains with CR fragments were named sinRm-CR and icdm-CR, respectively. The final strains with the single mutation were named sinRm and icdm, respectively, and the strain with *sinR* and *icd* double mutation was named sim. 

**Figure 5:**
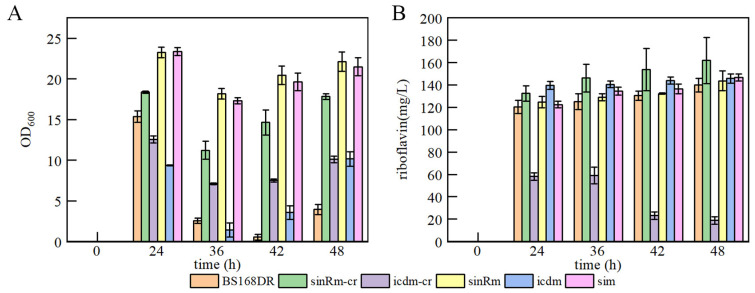
Batch fermentation by strains BS168DR and engineering strains in 500 mL shake flasks containing 80 mL of YP medium. (**A**) time course profile of cell growth; (**B**) riboflavin production during fermentation. The CR fragment was inserted into the middle of the target gene in sinRm-CR and icdm-CR strains, which were seen as the insertion knockout strains for fermentation to study the effect of the knockout mutation on the cells.

The results showed that the biomass of single mutation strain sinRm was greatly increased (at an OD_600_ of 23.24 at 24 h), and sinRm achieved a slightly higher riboflavin concentration than that of the control strain. The biomass of single mutation strain icdm remained at a relatively low level throughout the fermentation process, and its production was higher than that of the control strain. The change of sim was similar to sinRm with higher biomass and slightly higher production compared with the control strain (Figure 5A,B), which was consistent with the comparing results of U3 and S1 on the flask fermentation.

Someone investigated the strain with *icd* deletion and found that it had the characteristic of lower biomass. Through the simultaneous deletion of *icd* and *citZ*, namely citrate dehydrogenase, its biomass can be restored to the level of wild type, indicating that this phenomenon was due to the abnormal accumulation of citrate caused by *icd* mutation, which inhibited the formation of spores and growth [24]. Thus, the determination of the pH of the fermentation broth was carried out.

**Figure 6:**
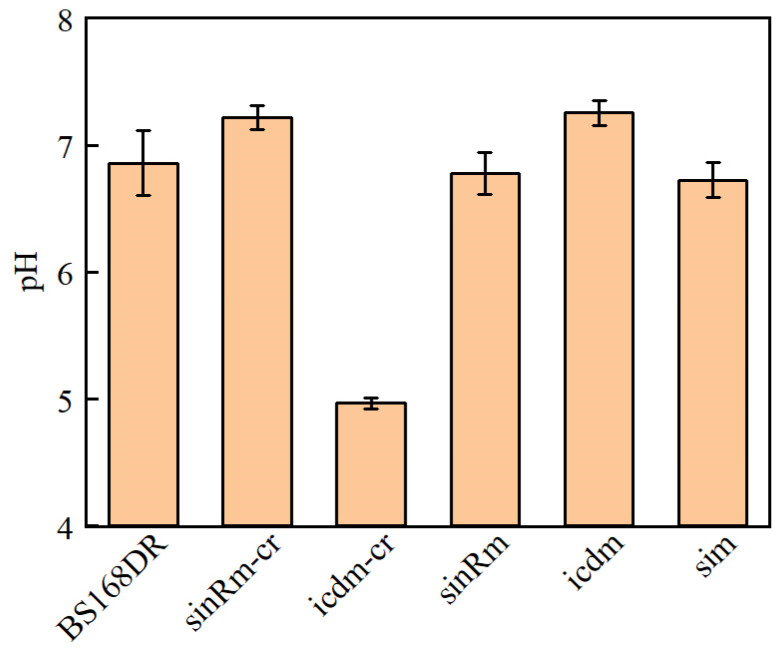
Comparison of pH between BS168DR and engineering strains. The pH was determined at 48 h of fermentation.

The results showed that the pH of the icdm-CR strain decreased significantly, while that of the icdm strain was slightly higher than that of the control strain (Figure 6). In addition, only the carbon source of icdm-CR was not consumed after 24 h. It indicated that the effect of icdm might be different from icdm-CR, which deserves to be further researched.

### 3.5. Biofilm Determination

**Figure 7:**
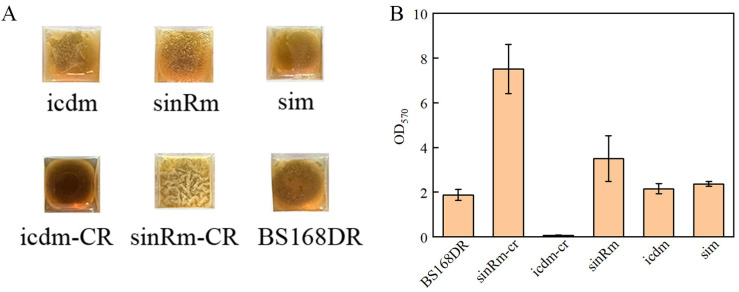
Comparison of biofilm morphology (**A**) and biofilm biomass (**B**) of BS168DR and engineering strains. The biofilm biomass was determined at 48 h of fermentation.

It was reported that *sinR* regulates biofilm synthesis. Compared with the wild-type strain, the biomass of the biofilm in the strain that knocked out the *sinR* gene increased by 2.8 times [27]. Thus, the biofilm determination was carried out. The results showed that the biomass of the sinRm biofilm was lower than that of the sinRm-CR strain, and the biofilm biomass of both strains was higher than that of the control strain (Figure 7A,B). Thus, it indicated that the effect of sinRm was to reduce the activity of SinR instead of completely inactivating it.

## 4. Discussion

In this study, the mutant strain U3 was obtained from the riboflavin-producing strain S1 by ARTP mutagenesis and screened by droplet-based microfluidics. Flask and fed-batch fermentation results showed that the production ability of U3 was stronger than that of S1. In the flask fermentation experiment, the production of U3 was 2138.32 mg/L at 46 h, while the control strains produced 2093.46 mg/L riboflavin. In the fed-batch fermentation experiment, the production of U3 and S1 was 24.3 g/L and 20.6 g/L, respectively, and the production of the U3 strain was 18% higher than that of S1. Anyway, the flask fermentation showed that the growth of U3 was also significantly increased than that of S1; the maximal OD_600_ of U3 reached 27.29 at 17 h while the OD_600_ of S1 was 19.24 at the same time. The mutation sites *sinR^G89R^* and *icd^D28E^* of U3 was identified by whole genome sequencing. To study the effect of the mutation sites on the growth and production, strains sinRm, icdm, sinRm-CR, icdm-CR, and sim were obtained by reverse introduction of the mutation site to BS168DR with a clear genetic background. The results showed that the characteristics of the sim strain compared with BS168DR were consistent with that of U3 compared with that of S1 on flask fermentation, which means higher biomass and slightly higher production. In addition, the strains with single mutation of *sinR* and *icd* had the characteristics of higher biomass and lower biomass compared with the control strain, respectively, and icdm produced more riboflavin than the control strain, while the production of sinRm was slightly higher than that of the control strain. Finally, the results indicated that the effect of *icd* mutation might be different from icdm-CR by measuring the pH of fermentation broth and residual sucrose, which deserved further research. The results of biofilm detection showed that the effect of *sinR* mutation was to reduce the activity of SinR, but the mutation did not completely inactivate SinR.

During the fermentation process, the residual glucose of U3 remained at a high level after the consumption of the initial glucose, and the glucose consumption per cell was higher than that of S1 when the glucose supplement was started. It showed that U3 is more sensitive to concentration changes of residual glucose than S1, resulting it had a demand for stable residual glucose at a high level. The fermentation conditions can be optimized to meet this demand, such as changing the glucose concentration of the fermentation medium or feeding medium, and different combinations of glucose concentration and feeding rate could be used as a variable for further optimization.

It was reported that a *sinR* mutant strain was screened according to the phenomenon that UDP-galactose is toxic to *B. subtilis* with *galE* deficiency. UDP-galactose is the synthetic precursor of EPS, and it was found that the content of EPS in the mutant increased significantly, thus relieving the toxicity [28]. The metabolism of UDP-galactose is relevant to that of glucose through galactose metabolism and glycolysis, so further study will focus on the UDP-galactose content of U3 to investigate the phenomenon of high residual glucose demand of U3.

It was reported that the deletion or overexpression of *sin* operon (*sinI* and *sinR*) would affect the late growth of the cell. The excessive *sin* operon gene product has an inhibitory effect on the production of specific protease (neutral protease and serine protease) for spore formation and late growth but has no obvious effect on the movement ability of the strain and can be used to adjust the late growth of the strain through modification [29]; It indicated that the mutation of *sinR* in U3, sinRm, and sim was the reason for higher biomass than that of control and other strains in flask fermentation at a late stage. The analysis of U3 and S1 off-gas in fed-batch fermentation indicated that the TCA cycle flux of U3 was reduced based on the characteristic of U3 carbon dioxide emission reduction. According to the results of verification fermentation, we judged that the effect of *icd* mutation was different from icdm-CR because of the detection results of fermentation broth pH and residual glucose. Thus, it indicated that the *sinR* mutation was the reason for the increasing biomass of sim, while the effect of *icd* mutation was not significant.

Except for *sinR* and *sinI*, there are many other genes that control biofilm synthesis. *YuaB* is a necessary gene in the biofilm-forming process, which is responsible for the hydrophobic layer on the surface of biofilms. Disruption of *yuaB* directly affects resistance to liquid wetting and gas penetration of the strains [30,31]. *FtsH* also played a significant role in biomass forming. The biofilm biomass of strain with mutant *FtsH was* greatly reduced [32]. Since both *sinR*, *sinI*, *yuaB*, and *FtsH* genes are genes that control biofilm synthesis, the mutations of *sinI*, *yuaB*, and *FtsH* genes might have a similar or synergistic effect with the mutations of *sinR* gene. Because the relationship between biofilm synthesis and biomass was not clear, the mechanism of increasing biomass can also be further analyzed by mutations of *sinI*, *yuaB*, *FtsH* genes, and other biofilm-related genes.

## Data Availability

The analyzed data presented in this study are included within this article. Further data are available upon reasonable request from the corresponding author.

## Supplementary Materials

The following supporting information can be downloaded at: https://www.mdpi.com/article/10.3390/microorganisms11041070/s1.
