# Supplementary material for: Characterization of a Riboflavin-Producing Mutant of Bacillus subtilis Isolated by Droplet-Based Microfluidics Screening
Source: Microorganisms. 2023 Apr 20;11(4):1070. doi: 10.3390/microorganisms11041070 (PMC10146818; doi:10.3390/microorganisms11041070)
Supplement: Supplementary file 1 [file microorganisms-11-01070-s001.zip › Supplementary Material.pdf]

Supplementary Material

1. Supplementary Table S1. Bacterial strains and plasmids used in this study

Name	Relevant phenotype	Source /reference
Strains		
B. subtilis 168	Wide-type strain, *trpC*2	Lab stock (from China General Microbiological Culture Collection Center)
BS168DR	neomycin resistance	Lab stock＆[12]
S1	Mutated from BS168DR, erythromycin and chloramphenicol resistance	Lab stock＆[11]
U1	Mutated from S1, erythromycin and chloramphenicol resistance	This study
U2	Mutated from S1, erythromycin and chloramphenicol resistance	This study
U3	Mutated from S1, erythromycin and chloramphenicol resistance	This study
U4	Mutated from S1, erythromycin and chloramphenicol resistance	This study
U5	Mutated from S1, erythromycin and chloramphenicol resistance	This study
U6	Mutated from S1, erythromycin and chloramphenicol resistance	This study
U7	Mutated from S1, erythromycin and chloramphenicol resistance	This study

U8	Mutated from S1, erythromycin and chloramphenicol resistance	This study
sinRm	BS168DR, *sinR**(G89R), neomycin resistance	This study
icdm	BS168DR, icd*(D28E), neomycin resistance	This study
sinRm-CR	BS168DR, *sinR**(G89R)-CR, chloramphenicol resistance	This study
icdm-CR	BS168DR, icd*(D28E)-CR, chloramphenicol resistance	This study
sim	icdm, *sinR**(G89R), neomycin resistance	This study

The resistance concentration used in this study were 60 μg/ml of neomycin, 20 μg/ml of erythromycin and 20 μg/ml of chloramphenicol respectively.

2. Supplemental Table S2. Primers used in this study

Name	Sequence (5’–3')
sinRm-UP1	CGTCGATCAAGGCTTAGGCT
sinRm-UP2	TCCAATTTTCGTTTGTTGAACTAATGGGTGCTTTAGTT GAAGACCATTCACTATCTAATTGACCATCGTATTCG
CR1	TCTTCAACTAAAGCACCCATTAGTTCAACA
sinRm-CR2	TCGATACCCTGGATGTCATCGCATCGCGAACCAATTT CTCCCATTCACTATCTAATTGACCATCGTATTCGGTTT CATGTTTATTCATTCAGTTTTCGTGCGGACT
sinRm-DN1	GAGAAATTGGTTCGCGATGCGAT
sinRm-DN2	GCTTTTGTGACGATCAGCAGC
icdm-UP1	CATCCCATGGCTGCTCTTCG

icdm-UP2	AATTTTCGTTTGTTGAACTAATGGGTGCTTTAGTTGA AGAGTTTGGTACGTTTAATACTCCGTTAGAGACTGTA A
icdm-CR2	CCGGTTCCTTCTCCTTCGATAAATGGGATAATCGGGT TGTTTGGTACGTTTAATACTCCGTTAGAGACTGTAAT TTTTTTATTCATTCAGTTTTCGTGCGGACTG
icdm-DN1	AACCCGATTATCCCATTTATCGAAGG
icdm-DN2	AGGACATTCCGACAATTCGCC

3. Part of the fluorescence microscopy images

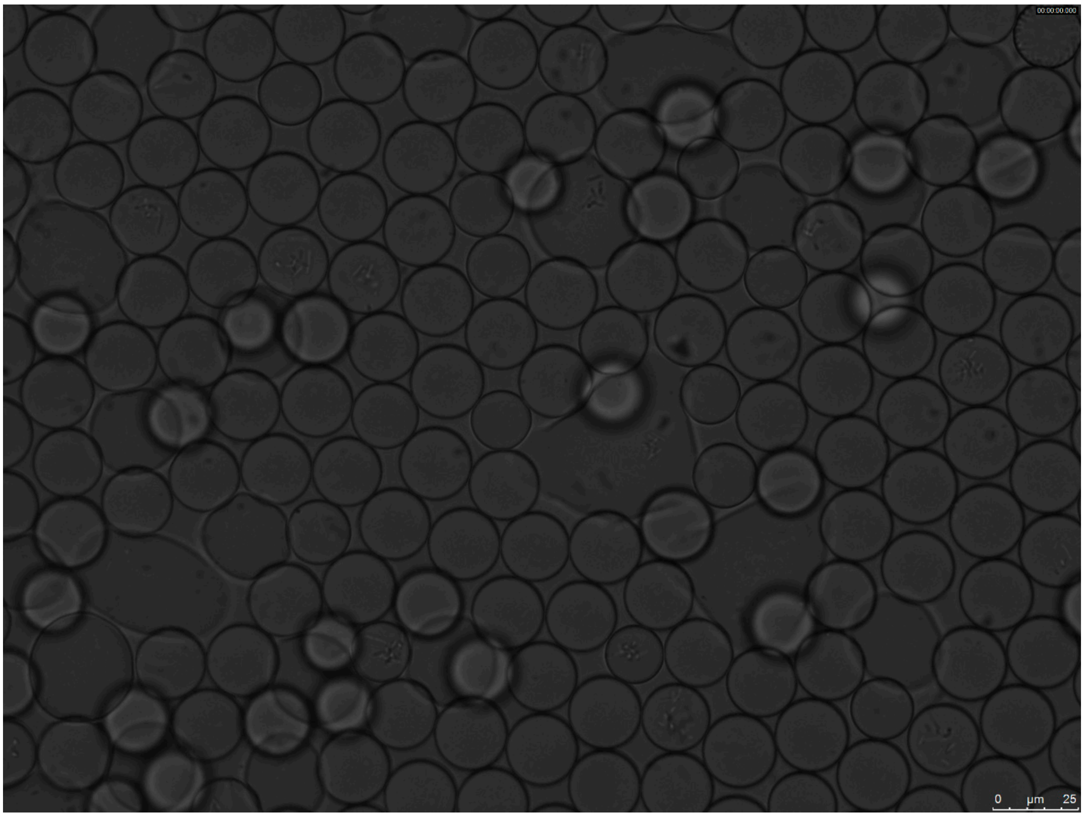

Figure S1 microscopy images at 4h

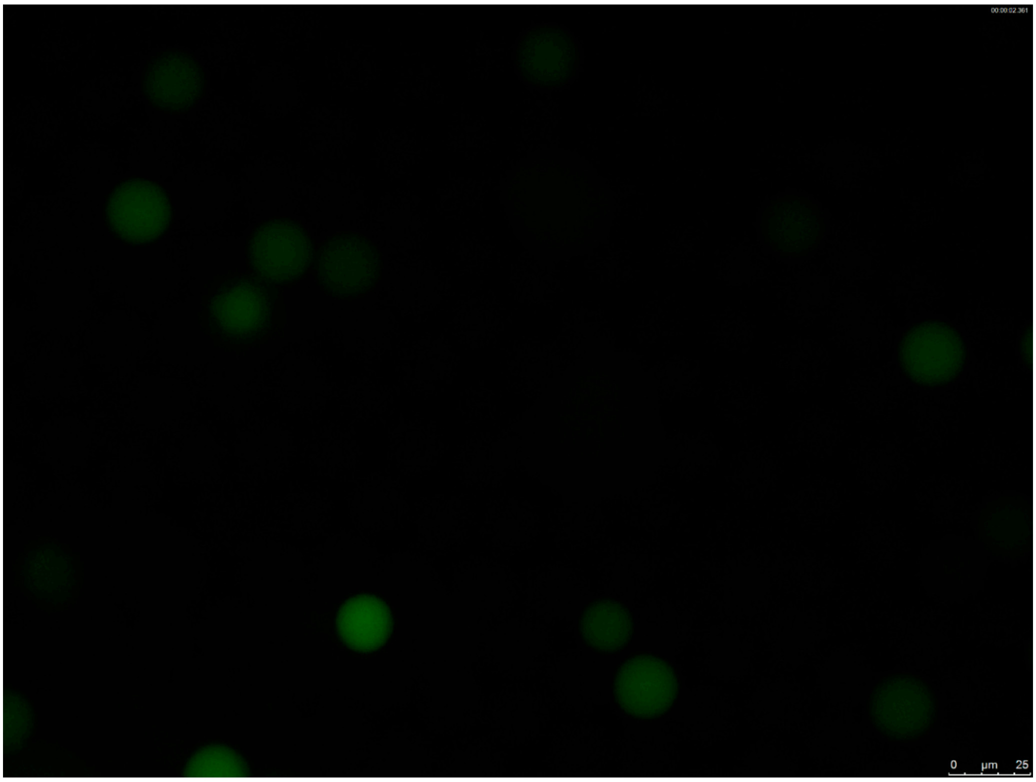

Figure S2 fluorescence microscopy images at 4h

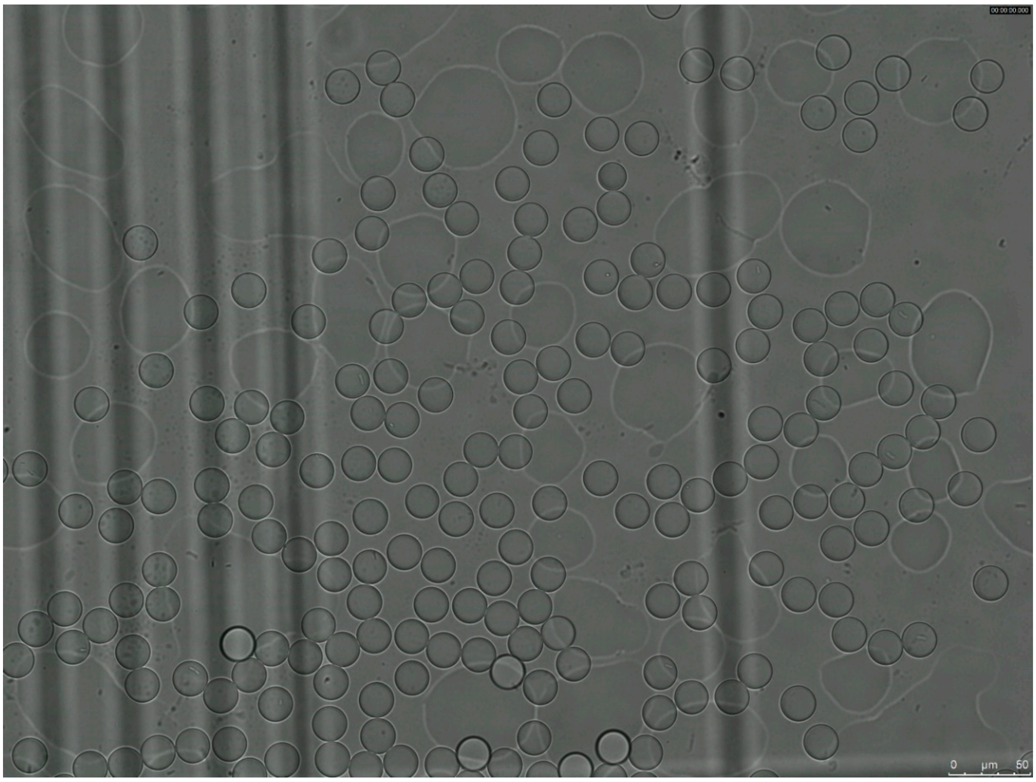

Figure S3 microscopy images at 0h

References

11. Shouying, F.; Miaomiao, X.; Yining, Z.; Chuan, L.; Ran, T.; Dawei, Z. Estabilishment and Application of High-throughput Screening Method of Riboflavin Industrial Strain. *Biotechnol. Bull.* **2020**, *36*, 47.

12. Dawei, Z.; Yuan, S.; Bin, Y.; Chuan, L. Engineering Strain of Bacillus subtilis with High Vitamin B2 Production, Its Construction and Application. CN Patent 113025550B, 10 September 2021.
